# Stimulus intensity determines experience-dependent modifications in neocortical neuron firing rates

**DOI:** 10.1111/ejn.12805

**Published:** 2014-12-26

**Authors:** Stanislaw Glazewski, Alison L Barth

**Affiliations:** 1 Department of Biological Sciences and Center for the Neural Basis of Cognition, Carnegie Mellon University4400 Fifth Avenue, Pittsburgh, PA, 15213, USA; 2 School of Life Sciences and Institute for Science and Technology in Medicine, Keele UniversityKeele, Staffordshire, UK

**Keywords:** cortical circuitry, glutamatergic neurotransmission, learning and memory, mouse, neocortex, plasticity

## Abstract

Although subthreshold inputs of neocortical sensory neurons are broadly tuned, the spiking output is more restricted. These subthreshold inputs provide a substrate for stimulus intensity-dependent changes their spiking output, as well as for experience-dependent plasticity to alter firing properties. Here we investigated how different stimulus intensities modified the firing output of individual neurons in layer 2/3 of the mouse barrel cortex. Decreasing stimulus intensity over a 30-fold range lowered the firing rates evoked by principal whisker stimulation and reduced the overall size of the responding ensemble in whisker-undeprived animals. We then examined how these responses were changed after single-whisker experience (SWE). After 7 days of SWE, the mean magnitude of response to spared whisker stimulation at the highest stimulus intensity was not altered. However, lower-intensity whisker stimulation revealed a more than 10-fold increase in mean firing output compared with control animals. Also, under control conditions, only ∽15% of neurons showed any firing at low stimulus intensity, compared with more than 70% of neurons after SWE. However, response changes measured in the immediately surrounding representations were detected only for the highest stimulus intensity. Overall, these data showed that the measurement of experience-dependent changes in the spike output of neocortical neurons was highly dependent upon stimulus intensity.

## Introduction

How does stimulus intensity alter the spike output and population responses in the somatosensory cortex? Unlike neurons in the periphery, whose firing rates are profoundly modulated by stimulus intensity, (Shoykhet *et al*., 2000) neocortical neurons typically fire at relatively low rates, usually at < 1 spike/stimulus (Barth & Poulet, 2012), and rate-coding schemes for individual neurons are implausible. Thus, it has been predicted that the overall spike output summed across large neuronal populations serves as an indicator of stimulus strength (Laughlin & Sejnowski, 2003). Increasing synaptic strength, either by state-dependent modulation or after experience-dependent plasticity (Finnerty *et al*., 1999; Petersen *et al*., 2003; Clem & Barth, 2006; Cheetham *et al*., 2007; Wen & Barth, 2011), could shift the stimulus–response curves for individual cells, enabling cells to spike at lower stimulus intensities. Remarkably, this has not been systematically investigated in the experimentally-tractable, well-studied rodent somatosensory system. Here we examine how stimulus strength is encoded by individual neurons in superficial layers of the mouse barrel cortex, and determine if this function was changed after altered sensory input, where animals retain only a single facial whisker.

We used single-unit recordings to isolate and record the activity of individual neurons directly, a method that is less invasive and has higher throughput than whole-cell recordings and can be maintained for enough time to test multiple stimulus conditions. By isolating neurons based upon their spiking response at high stimulus intensities, it is possible to determine how the response probability can be modulated as the stimulus intensity is changed.

First, we evaluated how a more than 30-fold change in stimulus intensity regulates firing output in control, whisker-intact mice, using a piezo-driven glass rod to deflect individual whiskers. Although such tuning curves have been referred to in previous studies (Armstrong-James & Fox, 1987; Boloori *et al*., 2010), their effects on the responses of individual cells within a population have not been systematically evaluated. Response amplitudes plateau as the stimulus intensity increases, suggesting that layer 2/3 neurons are not able to differentiate stimuli above a given threshold.

Second, we examined how plasticity induced by introducing an imbalance in whisker input [single-whisker experience (SWE)] can change the firing rates of neurons and the fraction of responding neurons across a range of stimulus intensities. Previous results have indicated that overall firing rates can be increased by this treatment (Diamond *et al*., 1993; Glazewski & Fox, 1996; Benedetti *et al*., 2009). This method enables us to examine whether the dynamic range of the responsive cell is altered. We found that SWE plasticity as measured in the spared, principal whisker barrel column is associated with an increase in the fraction of responding neurons at low stimulus intensities, as well as in the mean firing rate of these responsive neurons. The spike output was not significantly changed at high stimulus intensities after SWE, indicating that the dynamic range was specifically modified at the bottom end of the range.

## Materials and methods

### Animals

Recordings were made from 123 layer 2/3 neurons of 10 wild-type male C57/Bl6 mice (four control and six single-whisker animals) aged 39–52 days. All experiments were compliant with the UK 1986 Animals (Scientific Procedures) Act and with the European Union directive 2010/63/EU on the protection of animals for scientific purposes, were carried out with the approval of the Carnegie Mellon Institutional Animal Care and Use Committee and were compliant with the NIH directive for the use of animals in scientific research.

### Deprivation

All except a single whisker (D1 whisker remaining; SWE) were removed from one side of the mouse face for 7 days. All whiskers were removed from the other side of the face. This bilateral deprivation protocol was used because it generates robust plasticity in a short period of time (Clem & Barth, 2006; Glazewski *et al*., 2007). Whiskers were removed at the start of the deprivation period and every second day of the deprivation period by application of steady tension to the whisker base; this was performed under isoflurane anaesthesia. This deprivation technique does not affect vibrissae innervation (Li *et al*., 1995) and is described in detail elsewhere (Glazewski *et al*., 1998). This differs from prior studies in that there was no whisker regrowth period (which typically lasts 7–10 days). Therefore, only the spared whisker could be stimulated in SWE-treated animals.

### Anaesthesia and surgery

Animals were anaesthetised with urethane (1.5 g/kg of body weight) with trace amounts of acepromazine, injected i.p. All recordings were performed at stage III-3 anaesthetic level where a sluggish hind limb pinch withdrawal reflex and corneal blink reflex were present (Fox & Armstrong-James, 1986). Supplemental doses of urethane (10% of the initial dose) were administered to maintain the anaesthetic depth. Although urethane anaesthesia is associated with Upstate and Downstate fluctuations that can last ∽1 s each (Yassin *et al*., 2010), the 50 trial stimulus period effectively smoothed any short-term influence of these different states. Body temperature was monitored throughout the experiment and maintained at 37 °C using a rectal thermometer connected to a heating blanket (Harvard Apparatus). For recording, the skull was thinned over the barrel cortex with a dental drill (0–3 mm caudal to bregma and 2–4 mm lateral to midline). A small hole was made in the skull before each electrode penetration using a small hypodermic needle tip.

### Electrodes and recording

Custom-made single-barrel glass-insulated and sharpened carbon fibre microelectrodes were used to record extracellular potentials from the cortex (Armstrong-James *et al*., 1980). Electrodes were lowered perpendicular to the cortical surface. Electrode penetrations in control animals were made in layer 2/3 of various barrel columns representing the large facial whiskers in the posterior–medial barrel subfield, where responses are equivalent irrespective of the principal whisker (Glazewski *et al*., 1999). In whisker-deprived animals, electrode penetrations were made in layers 2/3 of the spared whisker barrel column (D1) and/or immediately adjacent barrel columns. Action potentials from single units were isolated using a Neurolog system (Digitimer, UK) and filtered between 0.7 and 7 kHz with a 50 Hz notch filter. Signals were amplified 2000×. Neurons that responded at <0.1 spikes/stimulus over 50 stimuli using the highest intensity stimulation were not included in the analysis. During recording, neurons were sampled at roughly 50 μm depth intervals.

### Cell isolation

For control and SWE animals, single units were recorded and a threshold and ceiling function was employed to isolate the cell from other responding cells during the recording. Spontaneous firing and also whisker deflection-driven firing were used to isolate a given cell, and responses were initially recorded at the highest stimulation intensity (in order to verify that there was indeed a responsive cell present). Spikes were isolated using a voltage window discriminator and spike events were stored as peri-stimulus times using CED 1401micro (CED, Cambridge, UK) and a computer running Spike2 software. The spike waveform shape was monitored during recording to ensure good spike isolation (Fig. S1). Post-stimulus time histograms and rasters were monitored on-line during stimulation.

### Stimulus

Stimulation consisted of a 10 ms vertical deflection of a single whisker contralateral to the recording site, delivered at 1 Hz (50 stimuli applied per stimulus intensity to account for the low fidelity of cortical responses) using a fast piezoelectric bimorph wafer attached to a lightweight glass capillary driven from a voltage source (DS-2, Digitimer) under the control of Spike2 software (CED). The stimulus intensity was varied using a calibrated piezoelectric device attached to a fine capillary glass rod, in order to move the whisker. The rod was positioned so that the whisker was gently contacting it at approximately 10 mm from the whisker base. For a particular whisker, stimuli were delivered from highest to lowest over a period of about 10 min. Although high-intensity stimuli were required to isolate a given unit (and so stimulus delivery could not be completely random), response constancy was confirmed by repeating the high-intensity stimulus at the end of the stimulus sequence. Using an optical probe to measure the stimulus properties (generous loan from Dan Simons, Pittsburgh, PA, USA), we determined that the stimulus amplitude measured in degrees of deflection was directly proportional to both the voltage used to deflect the whisker and the stimulus velocity. During the course of our experiments, we found that we needed to introduce even lower stimulus intensities to fully capture changes in response properties after SWE (0.04° and 0.014°). This intensity was not typically tested for control cells, as responses were nearly absent at 0.06°.

In our experiments, the stimulus velocity was tied to the stimulus amplitude, and we did not attempt to hold one parameter constant while systematically varying the other, as in some studies (Pinto *et al*., 2000; Stuttgen *et al*., 2006). Thus, changes in stimulus quality are described as changes in stimulus intensity, which encompasses both amplitude and velocity. The precise relationships between the stimulus amplitude and velocity used for these experiments are presented in Table1.

**Table 1:** Piezo-delivered stimulus intensities

Deflection (radial degrees)	Deflection (μm)	Velocity (μm/ms)
1.4	240	43.1
1.2	200	35.1
0.83	150	26.3
0.51	90	15.8
0.36	62	11.1
0.14	25	4.60
0.06	11	1.90
0.04	6.6	1.21

### Histology

At the end of each electrode penetration, a small lesion (1 μA, DC, 10 s, tip negative) was made in layer 4 to mark the location of each electrode penetration. After each experiment, the animals were deeply anaesthetised and perfused through the heart with 0.1 m phosphate-buffered saline followed by a 4% buffered solution of paraformaldehyde. The brain was removed, the cortex flattened as described previously (Strominger & Woolsey, 1987) and left overnight in buffered solution of 4% paraformaldehyde containing 30% sucrose. Sections of 40 μm thickness were cut tangentially to the surface of the flattened cortex using a freezing microtome and the tissue was reacted for cytochrome oxidase (Wong-Riley, 1979). Stained sections were later digitally analysed for lesion location and post-hoc correction of recording depths (i.e. laminar location). Cells <300 μm below the pial surface were classified as layer 2/3.

### Analysis

The magnitude of response (measured in spikes/stimulus) was calculated using Spike2 software (CED). The spontaneous activity of cells was collected in a 50 ms time window immediately prior to the stimulus. The evoked activity was collected from 3 to 53 ms after the onset of whisker deflection and any contribution of spontaneous activity to this evoked response was corrected by subtraction of the spontaneous activity from the firing rate following the stimulus. The vertical and horizontal distribution of cells recorded in the vicinity of the intact whisker representation was equivalent between experimental groups. Responses to the stimulation of principal (any whisker from control) or spared (D1 whisker for SWE-treated animals) and surround vibrissae were determined in cells pooled from each treatment group.

The response probability was calculated as the number of trials with a spike in the analysis window (3–53 ms window following the stimulus, as spikes occurring at latencies of 0–3 ms cannot be related to whisker stimulation) divided by the total number of trials (Benedetti *et al*., 2009). In this case, trials with single and multiple spikes were equivalent. The first spike latency was measured as the latency for the first evoked spike within this analysis window. For each cell, all first-spike latencies in 50 successive trials were averaged to generate a mean latency. The mean latencies of cells belonging to the same treatment group were averaged again.

### Statistics

Some datasets failed the Shapiro–Wilk test for normal distributions (especially at low stimulus intensities, because fewer cells maintained any spiking response), so non-parametric statistics were used for all comparisons. The Kruskal–Wallace test (a non-parametric anova) was used for multiple comparisons of more than two datasets. A two-tailed Mann–Whitney test (a non-parametric t-test) was used for direct comparisons of two datasets. Data are presented as mean and SEM, unless otherwise indicated.

## Results

### Stimulus–response tuning curves in control animals

**Figure 1:**
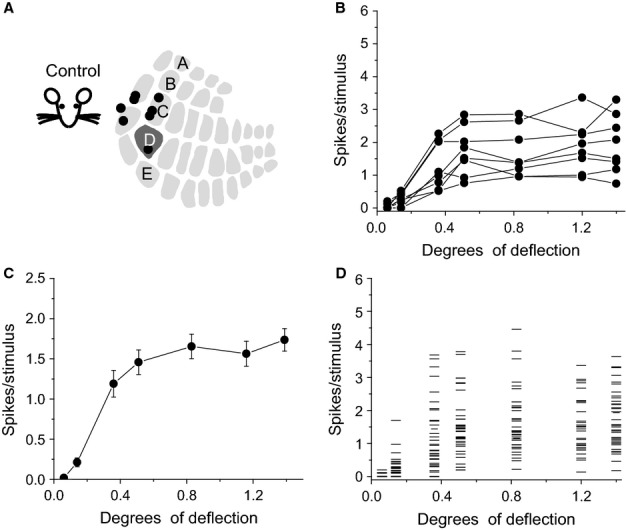
Stimulus-evoked firing rates plateau at higher intensities for the principal whisker responses in control, undeprived animals. (A) Distribution of electrode penetrations, marked by post-hoc electrical lesion and histologically confirmed for control mice. Barrel rows are labelled as A, B, etc. Because whiskers were undeprived, recordings from control animals were not necessarily directed to the D1 barrel column (dark grey). (B) Example responses for all cells from a single animal, where each point represents a 50 trial average response for each stimulus intensity (*n *= 12 cells). (C) Stimulus–response curves at multiple stimulus intensities for all cells from control animals (mean ± SEM), across an approximately 30-fold stimulus range (38 cells from four animals). (D) Distribution of mean spikes/stimulus for all cells across all stimulation intensities (38 cells from four animals).

Initially, we examined firing responses for a given cell across a more than 30-fold range of stimulus intensities (Table1). Interestingly, response magnitudes were similar at a broad range of the highest stimulus strengths (Figs1B–D and S2). For example, the highest stimulus intensity (1.4°) elicited on average 1.7 spikes/stimulus, but a stimulus that was nearly half that intensity (0.83°) also elicited 1.7 spikes/stimulus. Overall, the mean firing rates to the four highest stimulus intensities across a nearly threefold range were statistically indistinguishable (*P *= 0.7, Kruskal–Wallace test). The mean response curve for the population, measured across all intensities, could be well-fit by a single exponential (r^2^ = 0.98) and saturated at high intensities, with a small region where there was a roughly linear relationship between the stimulus intensity and spike output. At the low stimulus intensity (0.14°), the mean spike rate dropped to 0.019 spikes/stimulus, a value that takes into account cells that could no longer be triggered (i.e. showed 0 spikes/stimulus). If only those cells that fired are taken into account, the mean firing rate was 0.14 spikes/stimulus at this intensity. Deflection-triggered spike latencies in layer 2/3 neurons, measured by the timing of the first spike in the response, were significantly shorter at high than low stimulus intensity [high (1.4°), 10.4 ± 0.8 ms, *n *= 38 cells vs. low (0.14°), 20.0 ± 2.5 ms, *n *= 23 cells; *P* < 0.0001, Mann–Whitney test]. Overall, these data showed that the stimulus intensity was correlated with the evoked firing rate mainly in the middle of the stimulus range, and that, at the lowest intensities, the mean firing rates were reduced more than 10-fold.

### Reduced stimulus intensity leads to increased trial-to-trial spike failures

**Figure 2:**
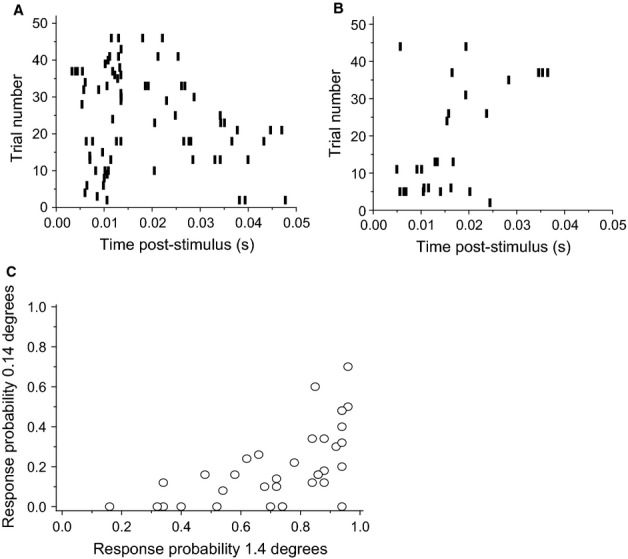
Reducing stimulus intensity increases spike failure rate in control animals. (A) Example of an isolated neuron response over 50 stimulus trials using the largest deflection (1.4°). Stimulus onset is at 0. Only 19 trials did not result in a spike. (B) The same cell responding to a 10-fold smaller deflection (0.14°), where the spike failure rate doubled (38/50 trials did not result in a spike). (C) Mean response probability for each control cell (calculated over 50 trials) compared at high and low stimulus intensities.

What factors control the decrease in spike output with decreasing stimulus intensity? Our previous work (Benedetti *et al*., 2009) showed that, for layer 2/3 neurons, most whisker deflection trials typically lead to a single spike within the post-stimulus detection window (50 ms), and in some fraction of trials neurons fail to spike at all. We hypothesised that failure rates would increase as stimuli became weaker. This was supported by the experimental findings. The data presented in Fig.2 show a representative control cell at 38% failure rate (19/50 trials) at the highest stimulus intensity, which subsequently increased to 76% (38/50 trials) at a lower stimulus intensity. For a given cell, failure rates slowly increased as the stimulus intensity decreased, until individual neurons failed to respond on any trial. The response rates were corrected for spontaneous firing (Fig. S3), which on average was very low (0.12 ± 0.02 Hz; *n *= 38 cells in four animals), and so these calculated failures were likely to represent a true lack of response.

### At lower stimulus intensities, most neurons stop responding

We next examined whether the stimulus intensity was correlated with sparseness, such that a larger group of neurons that are recruited at high stimulus intensities might be silent at low stimulus intensities. As the intensity of the whisker stimulus diminishes and trial-to-trial failure rates increase for a given cell, the cell would appear to drop out of the population. However, as the neuron was isolated using its spontaneous and evoked firing at the highest intensity stimulation level, we were confident that it was still present. This was confirmed in most cases by assessing the response to high-intensity stimulation at the conclusion of the stimulus series.

**Figure 3:**
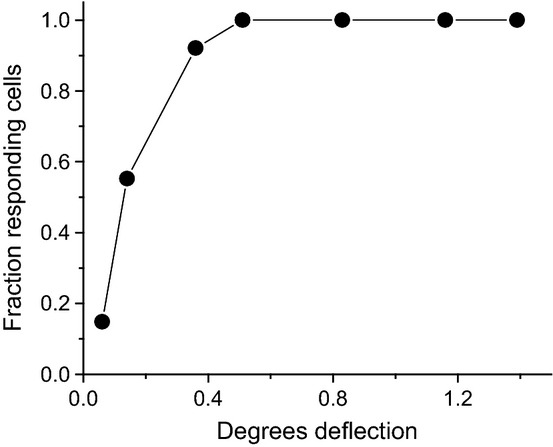
Population sparseness increases at low stimulus intensities in undeprived animals. The fraction of responding cells is reduced with decreasing stimulus intensity. By definition, all cells respond at high stimulus intensities, as this is how units were isolated. Response probability drops profoundly as stimulus intensity falls below 0.4° of deflection.

The population size was set as the number of neurons exhibiting > 0.1 spikes/stimulus at the highest stimulus intensity; by definition, 100% of neurons responded at the highest stimulus intensities. We then calculated the fraction of responsive neurons across all stimulus intensities (Fig.3). In control animals, this fraction was initially constant as the stimulation intensity decreased, even at levels where mean firing rates had modestly diminished. Thus, even at 0.51° deflection, 100% of neurons were still responsive (*n *= 38 cells). At lower stimulus intensities, the fraction of responding neurons dropped off sharply, with only 56% firing at 0.14° deflection and only 14.8% at 0.06°. Taken together with the analysis of trial-to-trial failures, these data indicated that stimulus strength was a critical variable in predicting whether a neuron will participate in the population of layer 2/3 neurons that encode whisker touch.

### Enhanced responses are only at low stimulus intensities after single-whisker experience

The relationship between stimulus intensity, response magnitude, and response probability for individual cells provided a metric to analyse whether the size of the responding ensemble can be changed by experience. If tactile information was encoded by a specific, determined subset of cells, we might predict that increased firing output after SWE would be observed as an increase in overall spike rates from this group of cells. Alternatively, sensory-driven plasticity might result in the recruitment of additional neurons to the response ensemble, altering the dynamic range of population responses as the stimulus intensity changes.

**Figure 4:**
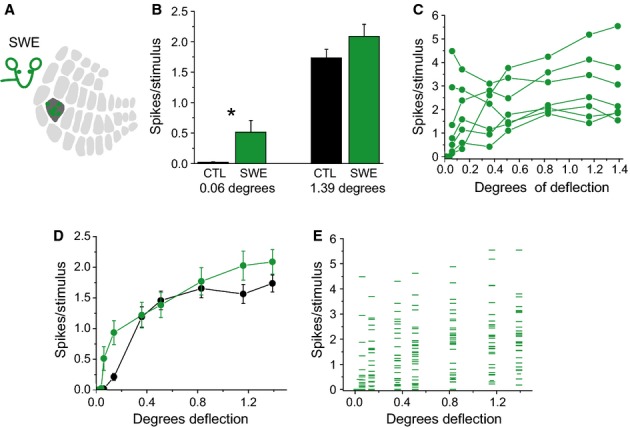
Plasticity in stimulus-evoked firing is most profound at low stimulation intensities. (A) Distribution of penetrations for SWE-treated animals, marked by post-hoc electrical lesion and histologically confirmed, were restricted to the D1 barrel column that represents the spared whisker. (B) Significant increases in mean firing output after SWE (bilateral deprivation) (*n *= 28 cells in six animals) are restricted to the lowest stimulation intensity, plotted with mean ± SEM values from control undeprived animals (CTL) (*n *= 28 cells in four animals; *P = 0.003, Mann–Whitney test). (C) Example responses for all cells from a single animal, where each point represents a 50 trial average for each stimulus intensity (*n *= 7 cells). (D) Stimulus–response curve for all cells from SWE-treated animals at multiple stimulus intensities (mean ± SEM) across an approximately 30-fold stimulus range (33 cells from six animals). Stimulus–response curve for control animals from Fig.1C is replotted for comparison. (E) Distribution of all cell responses from SWE-treated animals across all stimulation intensities (33 cells from six animals).

To determine how the stimulus intensity can influence the stimulus–response properties of layer 2/3 neural populations after sensory-induced plastic changes, we deprived animals of all but a single whisker from both sides of the face for 7 days. A significant increase in the firing rates of neurons in the spared whisker representation to high intensity stimuli was not observed at this timepoint (Figs4 and S4), unlike earlier studies that included a prolonged whisker regrowth period that may facilitate plasticity by extending the total time of SWE (Glazewski *et al*., 2007). However, there was a highly significant, almost 28-fold increase in mean firing rates for neurons at lower stimulus intensities where the mean firing rates were calculated from the entire sampled group, i.e. including cells that no longer fired to the 0.06° stimulus (Fig.4B). Even when cells that had ceased to respond to the stimulus were excluded from the average (reducing the *n* for this measurement), the mean firing rates were still sevenfold higher after the induction of whisker plasticity (control, *n *= 4 cells; SWE, *n *= 15 cells). Importantly, we did not observe any change in spontaneous firing rates in layer 2/3 neurons from SWE-treated animals (0.015 ± 0.02Hz; *n *= 34 cells in six animals; *P* = 0.3 vs. control, Mann–Whitney test) that might complicate our analysis (Fig. S5).

This specific increase in response output was manifested as a change in the shape of the stimulus–response curve across intensities, where response magnitude did not appear to plateau at higher intensities (Fig.4C–E) compared with control, as well as a leftward shift of the firing rate curve at lower stimulus intensities. Stimulus intensity response curves (mean spikes/stimulus) for selected individual neurons at the high, middle, and low end of the range are shown in Fig.4C.

Does the latency of the first evoked spike, a value that has been hypothesised to encode critical stimulus features (Chase & Young, 2007), change after SWE? We did not find this to be altered compared with control values at any stimulus intensity. The latency of the first spike in the response in neurons from SWE-treated animals was similar to that observed in control animals, at 9.89 ± 0.42 ms for high stimulus intensities (1.4°; *n *= 37 cells in six animals). As in control cells, the doubling of mean latency times at low stimulus intensity was also observed in SWE cells, where the first spike occurred at 18.16 ± 1.8 ms (0.14°; *n *= 25 cells in six animals). Thus, the potentiation of the response at low stimulus intensities was not accompanied by changes in the response latency, a finding that constrains the potential circuitry that might underlie this effect.

### Firing rate increase at low intensity correlates with failure rate decrease

**Figure 5:**
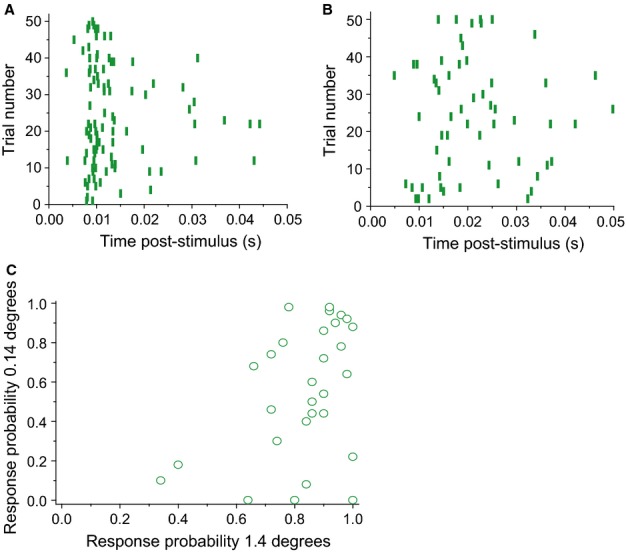
SWE reduces spike failure rates for weak stimuli. (A) Example of an isolated neuron response over 50 stimulus trials using the largest deflection (1.4°) in an SWE-treated animal. Stimulus onset is at 0. At the highest stimulation intensity, the spike failure rate for this cell is 10%; only five trials did not result in a spike. (B) The same cell at a low stimulation intensity (0.14°) showing a 46% spike failure rate. (C) Mean response probability for each SWE cell (calculated over 50 trials) compared at high and low stimulus intensities.

Following SWE, we found that overall trial-to-trial failures were inversely proportional to the stimulus intensity, i.e. as the stimulus intensity decreased failures rates increased. However, there was also a modest reduction in trial-to-trial failure rates after SWE-induced plasticity if compared with the undeprived control, and this was also found to be stimulus intensity-dependent (Fig.5A and B). This was most apparent at high stimulus intensities, where after SWE the mean trial-to-trial failure rate was 41.3% vs. 59.6% (*n *= 17) in control. The difference was, however, not large enough to significantly change the overall magnitude of response due to SWE. The shift in response probability was also observed at lower stimulus intensities, where the small group of control cells that maintained their response to very low-intensity deflections (0.06°) failed to respond on 84% of trials. The trial-to-trial failure rate for low-intensity stimuli was decreased to 75% after SWE.

Thus, the increase in mean spike output after the induction of plasticity stemmed at least in part from the increased response reliability of cells, an effect that was most pronounced at lower stimulus intensities.

### Whisker plasticity reduces population response sparseness

Stimulus detection in the primary somatosensory cortex is thought to be mediated in part by the overall number of neurons that are driven to spike (Houweling & Brecht, 2008; Huber *et al*., 2008). Thus, weaker whisker deflections might be more difficult to detect in part because the number of responsive neurons in the cortex is reduced, and their summated activity is less likely to propagate through the cortical column. Was there evidence for this in our dataset?

**Figure 6:**
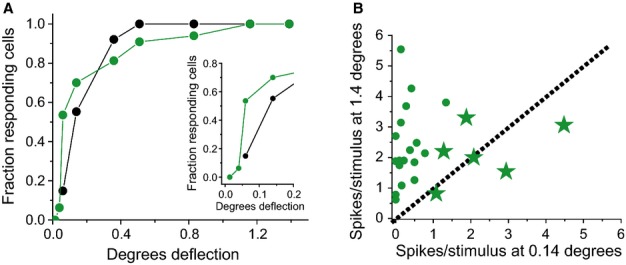
The size of the responding ensemble increases after whisker plasticity. (A) A comparison of the fraction of responding neurons vs. stimulus intensity shows a leftward shift in the curve after single-whisker-induced plasticity (green) compared with control cells (black) (replotted for comparison from Fig.3). (B) A cell-by-cell comparison of mean response at high (1.4°) and low (0.14°) whisker deflection. Stars highlight neurons that respond similarly or with a greater number of spikes to the low-intensity stimulus.

In control, undeprived animals, the fraction of responding cells indeed declined as the stimulus intensity was reduced, until at the lowest stimulus intensities, only a small fraction of neurons continued to display stimulus-evoked firing. This number was substantially altered by SWE (Fig.6).

The fraction of neurons responding in single-whisker-reared animals was increased at the lowest intensity measured for control animals (0.06°) to 54% (15/28 cells) compared with 15% in control (4/27 cells). Indeed, after SWE we found that we could still elicit firing in at least one cell with an even lower stimulus intensity of 0.04°. This is more than 30-fold lower than the maximal stimulus used to isolate the cell at the onset of recording, indicating a very broad dynamic range.

Small changes were also apparent at higher stimulus intensities, where we observed a small number of neurons that would ‘drop out’ with decreasing stimulus intensity. For example, in control animals, neurons isolated at the highest stimulus intensity continued to fire as the stimulus intensity was reduced about threefold (38/38 neurons maintained responsiveness from 1.4 to 0.51° deflection) and the mean firing rates were not significantly different. However, in SWE-treated animals, only 91% (30/33 neurons) were still firing at 0.51° deflection, and the overall firing rates appeared modestly reduced (Fig.6A) (control, 2.09 spikes/stimulus; SWE, 1.52 spikes/stimulus). As the comparisons between control and SWE-treated mice were not carried out within the same animal, it is difficult to determine whether this shift in the response curve is attributed to a different (larger) subset of responsive neurons in SWE-treated animals compared with controls, or could result from an altered dynamic range for intracortical inhibition.

The fraction of firing neurons after SWE slowly decreased in concert with stimulus intensity, but without the sharp reduction in firing observed in control animals. Interestingly, within-cell comparison of the response magnitude at low stimulation intensities (0.06°) vs. the response at the highest stimulation intensity (1.4°) showed that, in some cases, the mean firing response was virtually identical at both stimuli intensities (Fig.6B). This was never observed under control conditions (data not shown). Thus, the population of responding cells was made up of both cells that scale their response with stimulus intensity as well as neurons that have an all-or-none response, where firing is an indicator of stimulus presence but not necessarily stimulus intensity.

### Surrounding barrel columns change response only to high-intensity stimuli

**Figure 7:**
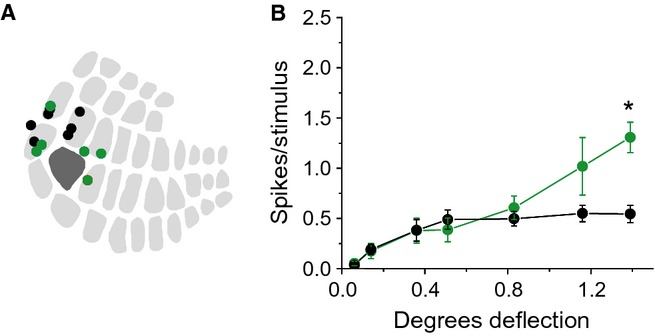
Responses of neurons in adjacent cortical columns are only recruited by high-intensity but not low-intensity stimulation after whisker plasticity. (A) Distribution of electrode penetrations, marked by post-hoc electrical lesion and histologically confirmed for control (black circles) and SWE-treated (green circles) animals. (B) The increase in firing output from stimulation of the adjacent (in control) or spared (D1) whisker in SWE-treated animals is only significant at the highest stimulation intensity (control, *n *= 17 cells in four animals; SWE, *n *= 27 cells in six animals; *P* = 0.0006, Mann–Whitney test).

Do neurons in adjacent, deprived barrel columns also display a shift in their firing output to different stimulation intensities of the spared whisker? To address this, electrode penetrations were made in barrel columns immediately adjacent to the spared barrel column. Responses were elicited by stimulation of the spared (D1) whisker. These responses are referred to as ‘surround’ responses, because they are generated by stimulation of an adjacent whisker. Typically, this elicits a weaker response than stimulation of the principal whisker. We found that increased firing rates elicited by stimulation of the spared whisker after SWE could only be observed using the highest intensity stimuli (Fig.7). Compared with control animals, where surround whisker responses recorded using the highest intensity stimulus elicited only 0.54 spikes/stimulus, neurons after SWE showed significantly larger evoked responses, with 1.3 spikes/stimulus. However, SWE did not increase the spike output to low-intensity stimuli. These findings showed that response potentiation in the spared barrel column did not necessarily lead to potentiation in surrounding columns, and constrain thinking about the specific neuronal circuits that must underlie this effect.

## Discussion

Here we present a systematic analysis of how stimulus intensity influences the firing responses of individual neurons in superficial layers of the somatosensory cortex, and describe how this response function can be changed by experience. In addition, we determined how altered sensory input via SWE could change the dynamic range of responsive neurons.

We found that layer 2/3 neurons in the anaesthetised mouse barrel cortex were driven by very small whisker deflections, consisting of only 1 radial degree or less of movement. At the top of this range, larger deflections neither increase the number of responsive cells nor enhance spike output, indicating a ceiling effect on the sensory response transformation in layer 2/3. Whisker-dependent plasticity was associated with the potentiation of responses specifically at the lowest stimulation intensities in the spared whisker representation, and specifically at the highest stimulation intensities in the immediately surrounding representations. These findings were not anticipated by prior studies that focused only on relatively high-intensity stimuli.

Finally, because responses from the same cell could be compared across stimulus intensities, these data show that the absolute number of responding cells becomes more sparse as the stimulus strength decreases in control animals. This sparseness was reduced in SWE-treated animals, as isolated neurons maintained their responsiveness across a significantly larger range of stimulus intensities.

### Single-unit recordings to assess stimulus-specific responses

The spiking responses across neurons in superficial layers have been well characterised in the rodent barrel cortex, where firing driven by whisker stimulation can be detected in only a subset of randomly distributed neurons (de Kock *et al*., 2007; O'Connor *et al*., 2010; Margolis *et al*., 2012). Both Ca^2+^ imaging and whole-cell recording studies have supported the notion that only a fraction of cells spike in response to a stimulus (Brecht *et al*., 2003; Kerr *et al*., 2007). However, even these methods have limitations that make it difficult to accurately assess the upper and lower limits that regulate the recruitment of neurons across a population. For example, although under optimal conditions Ca^2+^ imaging can detect single action potentials, it is most sensitive to bursts of action potentials (Kerr *et al*., 2007), but most spiking in the somatosensory cortex occurs as isolated action potentials (de Kock *et al*., 2007). Furthermore, although in theory the activity of all cells filled with the Ca^2+^ indicator can be monitored, in practice some cells do not show clear fluorescence changes, even when multiple spikes are present.

In addition, the delivery of Ca^2+^ indicators, whether through fluorescent indicators such as Oregon-Green BAPTA or via virally-transduced, genetically-encoded Ca^2+^ indicators, requires mechanical and/or chemical disruption that can influence the response properties and may alter signalling pathways that are critical for plasticity induction. Although the single-unit recording technique is biased toward the identification of neurons with a high basal firing rate, it is an efficient and minimally-invasive method to assess a broad range of stimulus attributes for an individual cell.

Single-unit recording methods do not capture all responsive neurons within a network as units are randomly isolated according to levels of spontaneous activity. It is possible that neurons with low levels of spontaneous activity were not detected. However, neurons with a different stimulus preference, e.g. a multiwhisker receptive field (Estebanez *et al*., 2012) or small differences in direction tuning (Andermann & Moore, 2006), should be captured based on their spontaneous activity. Because silent/unresponsive cells cannot be examined, it is impossible to determine the population size from which active cells are selected. We could, however, carry out within-cell comparisons of response probabilities of randomly isolated neurons across a range of high and low intensities.

### Single-whisker experience-induced changes in stimulus sensitivity

Selective whisker removal in the rodent leads to changes in the firing output of layer 2/3 neocortical neurons, a well-characterised form of experience-dependent plasticity (Glazewski & Fox, 1996; Glazewski *et al*., 1996; Benedetti *et al*., 2009). Previously, we have found that the removal of ipsilateral whiskers enhances plasticity, increasing the mean firing rate evoked by strong stimulation of the single spared whisker (Glazewski *et al*., 2007; Benedetti *et al*., 2009). However, unlike the current experiments, in previous studies adjacent whiskers were allowed to regrow for at least 7 days, effectively extending the deprivation period that may have enhanced responses to the spared whisker. In the current experimental paradigm, all but one whisker are removed from the mouse face for 7 days (SWE) without whisker regrowth. Under these conditions, the response potentiation of the spared whisker was not statistically significant at the highest stimulation intensities.

In contrast, we observed a profound and highly significant response potentiation at the lowest stimulation intensities. Indeed, during the course of our recordings from SWE-treated animals, we were required to readjust the lowest range of the stimulus in order to identify the threshold at which isolated neurons would cease to respond. The fraction of responsive cells for low-intensity stimuli was significantly larger than in control animals, and the mean spike output was larger, irrespective of whether we considered the entire population or just the cells that showed evoked spiking. These findings indicate that SWE can shift the response function of layer 2/3 neurons specifically for low-intensity but not high-intensity stimuli. This finding has implications for the way that specific neocortical circuits must be modified, so that SWE does not simply induce an overall increase in response output for all stimuli.

### Single-whisker experience-induced changes in surround vs. principal whisker responses

Principal whisker responses, i.e. the firing of neurons in the D1 column to D1 whisker stimulation, were specifically potentiated after SWE. However, previous studies have shown that neurons in surrounding barrel columns will exhibit stronger spared-whisker-evoked firing after SWE (Glazewski & Fox, 1996; Glazewski *et al*., 1996). Consistent with this, we also observed increased responses in surround columns, although this increase was only apparent for the highest stimulation intensity. This is intriguing, given that these responses were specifically not affected within the spared column. Thus, the SWE-dependent increase in spike output in surround columns is unlikely to be generated from more spiking of spared-column layer 2/3 neurons. Further dissociation of intracolumnar potentiation from intercolumnar potentiation can also be observed by comparing spiking responses at the lowest stimulus intensities; these were enhanced in the spared column but unchanged in the surrounding columns. Thus, we propose that the neocortical circuitry underlying the potentiation of responses in spared and surround columns may be different.

It is possible that the changes that we observe might be due to alterations in firing at earlier stages of processing, such as layer 4 or the thalamus. Further experiments will be required to identify whether layer 2/3 inherits, generates, or amplifies changes that might be occurring at other levels in this sensory pathway.

### Ensemble size is increased after single-whisker experience

Although our recording method did not enable us to establish the fraction of responsive neurons within the barrel column, as we could not ‘see’ silent cells with our recording electrode, it did allow us to determine how the size of the responsive population could be modulated by stimulus intensity. In control animals, a more than 20-fold decrease in stimulus intensity (from 1.30 to 0.14°) led to ∽85% reduction in the size of the responsive ensemble. In contrast, the same decrease in stimulus intensity after SWE led to only a 50% reduction in the size of the response ensemble. Although there may be a larger number of cells in the responsive population after SWE, a possibility that is consistent with prior studies (Margolis *et al*., 2012), we expect that the total number of responsive cells is increased for low-intensity stimuli after SWE. This is consistent with experimental data indicating that some layer 2/3 neurons normally receive principal-whisker-derived synaptic input that may be subthreshold, and that these synaptic inputs can be potentiated by SWE, thus increasing the size of the responsive population. These findings suggest that sparseness can be modulated by experience-dependent plasticity.

### Stimulus intensity is reflected in spike latency

Does latency convey information about stimulus strength in this dataset? The first-spike latency was dependent upon stimulus intensity, where the smallest intensity stimulus triggered layer 2/3 spikes at ∽10 ms later than larger intensity stimuli, which was observed in both control and SWE-treated animals. The dependence of the first-spike latency on stimulus intensity across conditions suggests that this information may at least partially encode stimulus strength, a possibility that has been suggested by some models (Storchi *et al*., 2012).

The difference in spike latency for high-intensity and low-intensity stimuli suggests different intracortical mechanisms for generating suprathreshold responses, where recurrent activity within the cortical column may be critical for initiating spikes for low-intensity deflections. As the mean latency for low-intensity stimuli was identical in control and SWE animals, it suggests that the same circuitry might be responsible for spike generation. The fact that spike generation is more likely after SWE is consistent with the specific strengthening of this circuitry, e.g. between layer 2/3 neurons but not inputs from the thalamus or from layer 4. Previous studies have indicated that excitatory pathways within superficial layers of the cortex can be potentiated by selective whisker activity (Clem & Barth, 2006; Cheetham *et al*., 2007; Wilbrecht *et al*., 2010; Wen & Barth, 2011; Jacob *et al*., 2012) in both young and adult animals.

### Perceptual changes after single-whisker experience

Layer 2/3 neurons are highly responsive to very small whisker deflections, exhibiting saturating responses at <1 radial degree of movement. This finding is consistent with the biological specialisation of this apparatus for the detection of very small whisker movements or fine textures (Jadhav *et al*., 2009). We find that the spike output is related to the stimulus intensity only within a narrow stimulus range, determined to be from 0.1 to 0.8 radial degrees of movement. Above a certain stimulus threshold, the responses saturate and below a certain threshold most neurons stop firing.

The rather narrow dynamic range for barrel cortex neurons is quite unusual, as in other sensory systems the stimulus intensity, such as increased visual contrast, increases the spike output across a broad range of firing frequencies, even in anaesthetised animals (Albrecht & Hamilton, 1982). The fact that this does not occur in the superficial layers of the somatosensory cortex indicates that, above a certain threshold, the stimulus intensity may not be a stimulus feature that must be accurately encoded by firing rates in these neurons. This is in contrast to neurons in the trigeminal ganglion, which can increase their firing output with increasing stimulus intensity across a broader range (Shoykhet *et al*., 2000).

The saturation of responses at high stimulus intensities indicates that, above some threshold, the intensity information is either not encoded by layer 2/3 neurons, or it is encoded by some different parameters than those investigated here (i.e. direction of whisker deflection, mean spikes/stimulus, first-spike latency, or spiking in surrounding barrel columns), and suggests that layer 2/3 of the neocortex may not be essential in the discrimination of more intense stimuli. However, other neurons within or outside the cortical column (e.g. in the colliculus; Cohen & Castro-Alamancos, 2010) may show a broader dynamic range of firing output at larger stimulus intensities. These possibilities should be addressed in awake, behaving animals, where the spike output is not suppressed by anaesthesia ((Barth & Poulet, 2012) and examined in multiple neocortical layers and brain areas. Several recent studies indicate that rodents can detect and discriminate stimuli that vary by small increments (Celikel & Sakmann, 2007; Adibi *et al*., 2012); such behavioural paradigms will be appropriate for the analysis of this hypothesis.

Taken together, these data provide a benchmark for how stimulus intensity can be transformed into spike output across a population of neocortical neurons, and demonstrate that this function can be altered in highly specific ways during experience-dependent plasticity.

## Abbreviations

SWE: single whisker experience

## Supporting Information

**Fig. S1**. Isolation of extracellularly-recorded spikes in L2/3 neurons. **Fig. S2**. Boxplot presentation of spikes/stimulus response for control layer 2/3 cells presented in Figure 1. **Fig. S3**. 50-trial sequence showing an isolated cell with relatively high spontaneous activity (grey box) prior to the onset of whisker deflection at 0s. **Fig. S4**. Boxplot presentation of spikes/stimulus response for SWE layer 2/3 cells presented in Figure 4. **Fig. S5**. Spontaneous activity is not changed after SWE.
