# Supplementary material for: Stimulus intensity determines experience-dependent modifications in neocortical neuron firing rates
Source: Eur J Neurosci. 2014 Dec 26;41(4):410–9. doi: 10.1111/ejn.12805 (PMC4331261; doi:10.1111/ejn.12805)
Supplement: Supplementary file 1 — Fig. S1. Isolation of extracellularly-recorded spikes in L2/3 neurons. Fig. S2. Boxplot presentation of spikes/stimulus response for control layer 2/3 cells presented in Figure 1. Fig. S3. 50-trial sequence showing an isolated cell with relatively high spontaneous activity (grey box) prior to the onset of whisker deflection at 0s. Fig. S4. Boxplot presentation of spikes/stimulus response for SWE layer 2/3 cells presented in Figure 4. Fig. S5. Spontaneous activity is not changed after SWE. [file ejn0041-0410-sd1.pdf]

Supporting Information:

Stimulus-intensity determines experience-dependent modifications in neocortical neuron firing rates

Stanislaw Glazewski and Alison L. Barth

Inventory of Supplemental Figures:

Figure S1. Isolation of extracellularly-recorded spikes in L2/3 neurons.

Figure S2. Boxplot presentation of spikes/stimulus response for control layer 2/3 cells presented in Figure 1.

Figure S3. 50-trial sequence showing an isolated cell with relatively high spontaneous activity (grey box) prior to the onset of whisker deflection at 0s.

Figure S4. Boxplot presentation of spikes/stimulus response for SWE layer 2/3 cells presented in Figure 4.

Figure S5. Spontaneous activity is not changed after SWE.

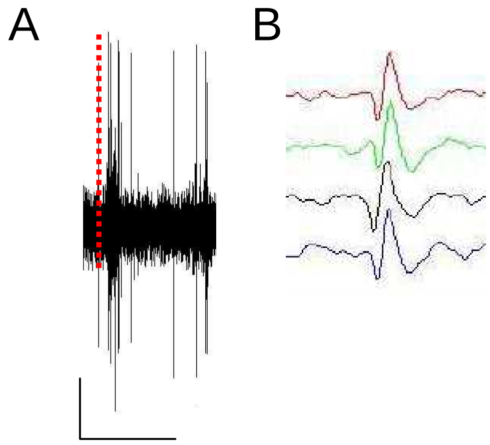

Figure S1. Isolation of extracellularly-recorded spikes in L2/3 neurons. A) Typical example of a response, where spikes are clearly visible above the noise. Multiple spikes are picked up by the electrode before an amplitude minimum and maximum cut-off window is applied. Dotted red line=time of whisker deflection onset. Scale, 50 μV, 500 ms. B) Example of 4 isolated spikes after application of the window discriminator from the cell in (A). This method can extract and calculate response times of individual spikes with similar waveforms.

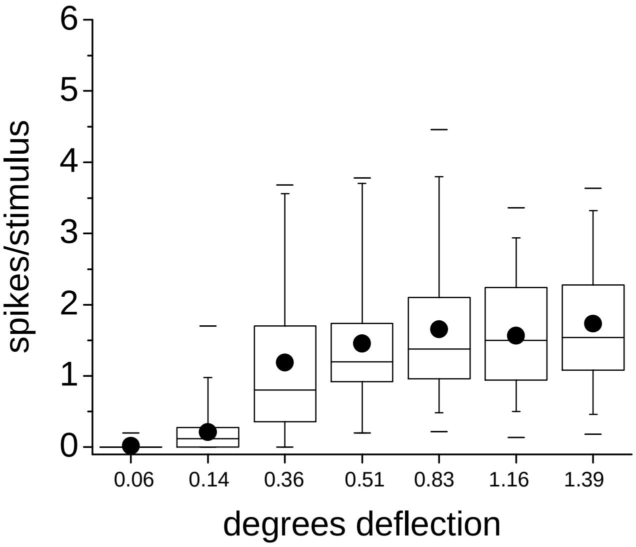

Figure S2. Boxplot presentation of spikes/stimulus response for control layer 2/3 cells presented in Figure 1. Mean value is shown as circle; external horizontal lines are maximum and minimum values. Box represents lower quartile and upper quartile. Middle line is median. Whiskers represent 5-95% percentile.

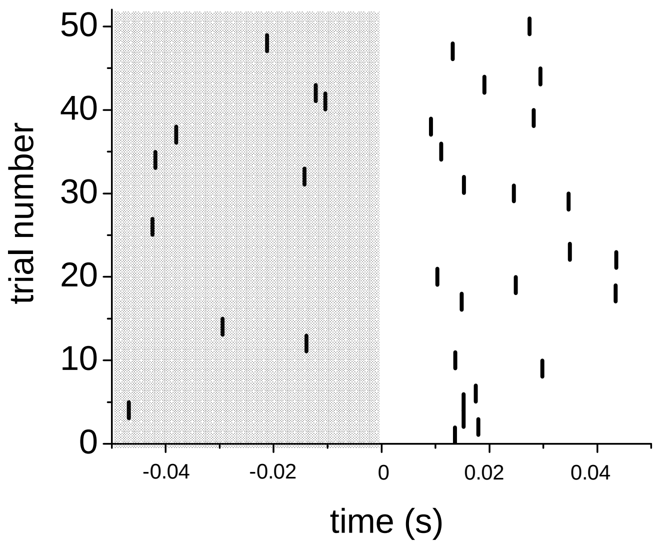

Figure S3. 50-trial sequence showing an isolated cell with relatively high spontaneous activity (grey box) prior to the onset of whisker deflection at 0s. In 10/50 trials, a single spike occurred in the 50 ms window preceding the stimulus. Thus, 10 spikes would be subtracted from the 23 spikes observed after the stimulus, for an evoked spike rate of 0.26 spikes/stimulus.

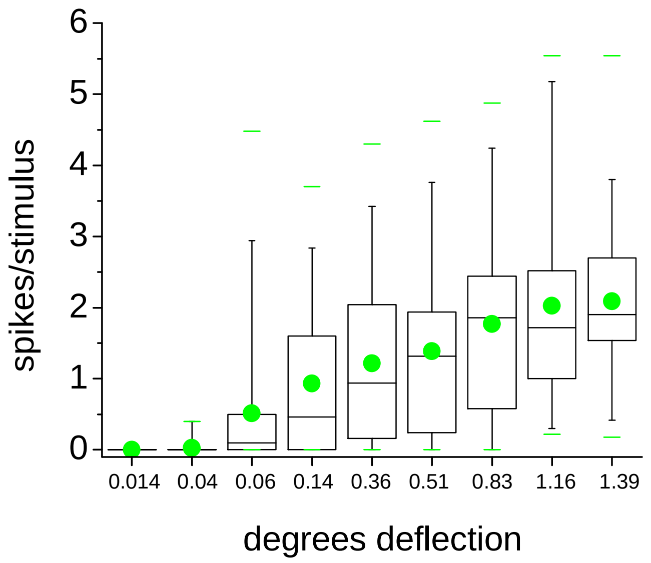

Figure S4. Boxplot presentation of spikes/stimulus response for SWE layer 2/3 cells presented in Figure 4. Mean value is shown as circle; external horizontal green lines are maximum and minimum values. Box represents lower quartile and upper quartile. Middle line is median. Whiskers represent 5-95% percentile.

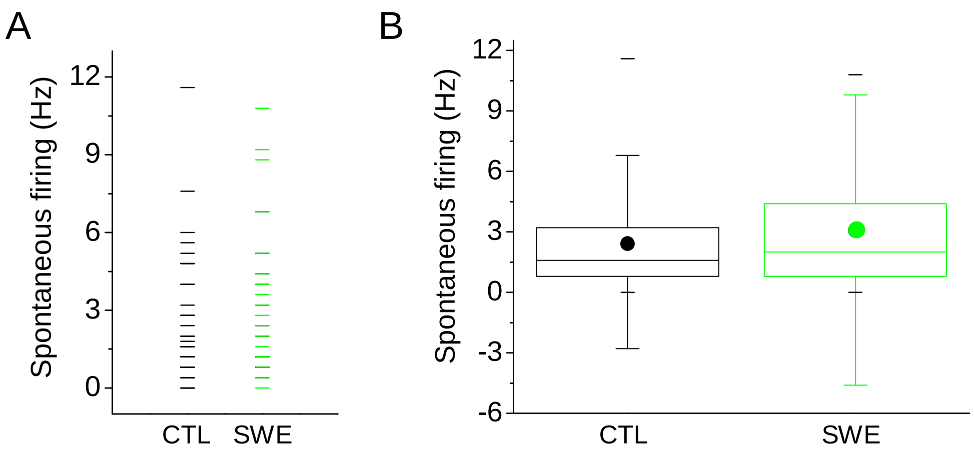

Figure S5. Spontaneous activity is not changed after SWE. A) Scatter plot of values from layer 2/3 control (CTL) and SWE cells. B) Boxplot presentation of (A).
